# Do we need aerobiological air monitoring in desert climates? The Qatar experience

**DOI:** 10.5339/qmj.2022.fqac.28

**Published:** 2022-04-01

**Authors:** Dorra Gharbi, Maryam A. Al-Nesf, Maria del Mar Trigo

**Affiliations:** ^1^Allergy and Immunology Division, Department of Medicine, Hamad Medical Corporation, Doha, Qatar E-mail: dorragharbi@yahoo.fr; ^2^Department of Botany and Plant Physiology, University of Malaga, Campus de Teatinos, Malaga, E-29071, Spain

**Keywords:** aeroallergens, aerobiological monitoring, allergy therapy

## Abstract

Knowledge about diurnal, seasonal, and annual fluctuations in airborne pollen and fungal spores in any geographical area is essential for effective diagnosis and treatment of allergies. Aerobiological analysis enables the detection of airborne pollen and spores, thus providing information on plant phenology, plant distribution, related diseases, and the risks for some species in terms of allergies. Although pollen and fungal spores have been widely studied as aeroallergens throughout the world, not much is known about the biological aerosols in countries with a desert environment; and these could be present in much higher concentrations than expected. Arid desert regions (including the region surrounding the Arab Gulf), characterized by hot weather, poor soils, and low biological productivity, have typically been neglected when building ambitious biomonitoring networks for the large-scale monitoring of biological particles; however, few studies in Kuwait, Saudi Arabia, and recently Qatar have aimed to delineate the various botanical families that contribute to inhalant allergens in this region.

Understanding the aerobiological features of countries with hot and desert climates may better prepare healthcare providers to assist patients with allergic rhinitis. It may be argued that one of the reasons why aerobiologists have only recently turned their attention to the state of Qatar investigating how pollen and fungal spore records could contribute to evaluating the correlation between different pollen conditions and allergy symptoms.

The first aerobiological network of Qatar was monitoring (2017–2020) the atmospheric pollen concentrations of Doha and Al Khor to determine the association between the possible risk of respiratory allergies and the distribution of certain species throughout the region. In the Qatari database, more than 25 native taxa have been recorded, up to 50% of which can be considered allergenic. This includes Amaranthaceae and Poaceae pollen among the major aeroallergens causing allergy symptoms in Qatar. Our study has confirmed a statistically significant association between Amaranthaceae and asthma and allergic rhinitis. To summarize, it is worth considering aerobiological monitoring in desert climate regions when assessing the effectiveness of pollen allergy therapy and planning prevention methods for patients.

